# Enhancing the functionality of a microscale bioreactor system as an industrial process development tool for mammalian perfusion culture

**DOI:** 10.1002/bit.26946

**Published:** 2019-02-20

**Authors:** David J Sewell, Richard Turner, Ray Field, William Holmes, Rahul Pradhan, Christopher Spencer, Stephen G Oliver, Nigel KH Slater, Duygu Dikicioglu

**Affiliations:** ^1^ Department of Chemical Engineering and Biotechnology University of Cambridge Cambridge UK; ^2^ BioPharmaceutical Development Division MedImmune Cambridge UK; ^3^ Cambridge Systems Biology Centre University of Cambridge Cambridge UK; ^4^ Department of Biochemistry University of Cambridge Cambridge UK

**Keywords:** Chinese hamster ovary, gravity cell settling, microscale process development, perfusion reactors, upstream processing

## Abstract

Without a scale‐down model for perfusion, high resource demand makes cell line screening or process development challenging, therefore, potentially successful cell lines or perfusion processes are unrealized and their ability untapped. We present here the refunctioning of a high‐capacity microscale system that is typically used in fed‐batch process development to allow perfusion operation utilizing in situ gravity settling and automated sampling. In this low resource setting, which involved routine perturbations in mixing, pH and dissolved oxygen concentrations, the specific productivity and the maximum cell concentration were higher than 3.0 × 10^6^ mg/cell/day and 7 × 10 ^7^ cells/ml, respectively, across replicate microscale perfusion runs conducted at one vessel volume exchange per day. A comparative analysis was conducted at bench scale with vessels operated in perfusion mode utilizing a cell retention device. Neither specific productivity nor product quality indicated by product aggregation (6%) was significantly different across scales 19 days after inoculation, thus demonstrating this setup to be a suitable and reliable platform for evaluating the performance of cell lines and the effect of process parameters, relevant to perfusion mode of culturing.

## 1 INTRODUCTION

Since the approval of the first licensed monoclonal antibody (mAb) in 1986, the demand for mAbs as therapeutic agents has developed into a multi‐billion dollar per year market (Liu, 2014). Process optimization for large manufacturing facilities based on fed‐batch cell cultivation allowed sufficient productivity of high product quality mAbs (Birch & Racher, 2006). On the other hand, continuous bioprocessing employing perfusion bioreactors is utilized in the production of molecules that proved to be challenging to express or recover. The low bioreactor residence times associated with continuous production systems, and the continuous removal of potentially toxic by‐products, allows cells to reach high cell concentrations and improves total functional production. Currently, continuous production is widely accepted as beneficial for the manufacture of products such as infliximab (Remicade) that exhibited particularly low titres (B. Kelley, 2009), and haemoglobin factor VIII (ReFacto), which required minimal residence times in the bioreactor (Vogel et al., 2012).

As the demand for therapeutic mAb production increased, key improvements were made in the areas of safety, efficacy, and quality (Dimitrov, 2012), among which the utilization of scale down systems to investigate process robustness played an important role (Kochanowski & Malphettes, 2013). Automated single use microscale bioreactor systems were evaluated as a model for large scale fed‐batch bioprocessing systems (Hsu, Aulakh, Traul, & Yuk, 2012; Moses, Manahan, Ambrogelly, & Ling, 2012; Nienow et al., 2013). Microscale bioreactors were reported to shorten the timeline towards commercial production by allowing multifactorial process optimization, in line with the current Quality by Design guidelines (FDA, [Link]). pH and dissolved oxygen (DO) could be accurately controlled both in microscale and in large scale (Rameez, Mostafa, Miller, & Shukla, 2014). Informing process control at large scale involves an extensive understanding of the physical characteristics and limitations of the system investigated at microscale (Nienow et al., 2013).

With the increasing interest in continuous bioprocessing, specific industrial applications are now assessed on a case by case basis (Shukla, Wolfe, Mostafa, & Norman, 2017), from an operational, environmental, and economic stance (Pollock, Ho, & Farid, 2013). The consideration of the option of a continuous process necessitates the comprehensive process development and optimization be carried out within compatible time scales and operational complexity to that established for fed‐batch processes, whereby microscale systems prove vital (Birch & Racher, 2006). Commercial fed‐batch microscale system performance has been reported to demonstrate sufficient scalability to bench scale (Hsu et al., 2012). Microscale systems operated at continuous mode are yet to be evaluated to ascertain the scalability of the operation with regard to process parameters such as maximal cell concentration supported, productivity and product quality attributes.

To achieve very high cell concentrations, perfusion processes require a cell retention device. Such devices exploit physical properties, such as cell size (filtration) or density (gravity settling or centrifugation) and have been evaluated for their performance at increasing scales (Voisard, Meuwly, Ruffieux, Baer, & Kadouri, 2003). Adaptation of existing fed‐batch microscale cell culture systems to continuous cell culturing applications is a relatively recent avenue for perfusion process development. Due to the highly engineered nature of microscale systems, it is difficult to repurpose them for perfusion by retrofitting a cell retention device. Microtitre plate based systems such as SimCell platform (Seahorse; Legmann et al., 2009) and BioLector Pro (m2p labs; Blesken, Olfers, Grimm, & Frische, 2016) offer high experimental capacity. These systems, however, would require significant operator interaction to mimic perfusion through an adapted cell retention methodology. Alternative small scale perfusion models for media development were also evaluated (Du et al., 2015; Huang, Lin, & Yang, 2015); these, however, were not able to measure and control process parameters in real time to allow extensive bioprocess development activities to be conducted.

More recently, microscale systems have been developed that were specifically designed to operate in perfusion mode (Mozdzierz et al., 2015) but they have not yet been evaluated within the context of mammalian expression systems and, as novel applications, inherently will require significant economic and technical investment from the end user point of view.

Gravity cell settling within the culture vessel was proposed as a method that allows perfusion without the requirement to retrofit a cell retention device. Earlier studies conducted using traditional gravity settling devices (Searles, Todd, & Kompala, 1994) can successfully be adopted in the design of a microscale cell settling method. An early application using this strategy (Goletz, Stahn, & Kreye, [Link]) and several industrial investigations (BioMarin Pharmaceutical, 2017; MilliporeSigma, 2017) highlighted an emerging interest in adopting this method, although no specific reports exist on a thorough comparative evaluation of this adapted system.

Here, the development and comparative evaluation of a microscale bioreactor system operating under pseudo‐perfusion mode, which utilizes gravity cell settling, is presented. The operational capacity of microscale bioreactor systems (both the 24‐ and 48‐vessel variants) and the functionality of the robotic liquid handling arm are explored to design an effective perfusion mimic microscale process. The transient effects of temporary interruption of agitation and gas sparging on the culture vessels with regard to settling time is investigated to assess the operational feasibility of this high‐capacity system. The productivity of a mammalian cell line expressing a mAb, and the product quality under operation in microscale perfusion mode are compared to their cognate values in the commonly used bench top perfusion reactor operating on alternating tangential flow (ATF) with hollow fibre filter for cell retention.

## 2 MATERIALS AND METHODS

### 2.1 Growth environment and cultivation conditions

A Chinese hamster ovary (CHO) cell line expressing a high concentration of bispecific mAb was used in this study. Two animal component free proprietary media compositions were used; one developed specifically for passaging cultures (basal), and another developed for supporting high cell concentrations associated with perfusion systems (enriched). The cells were revived from a frozen cell stock and sub‐cultured twice weekly in vented shake flasks (Corning, Corning, NY) containing basal medium in a humidified orbital shaker incubator maintained at 37°C with ambient 5% CO_2_ content.

### 2.2 Microscale and bench top perfusion process operation regimes

In Experiment 1, two 7 L stirred‐tank reactor (STR) bench scale (Chemglass, Vineland, NJ) vessels and 24 15‐ml microscale bioreactors (ambr 15; Sartorious, Gottingen, Germany) were inoculated from the same source, and cells were cultured in basal media, with a starting volume of 3.5 and 15 ml, respectively. Upon the initiation of perfusion, the working volumes are adjusted to 3 and 10.7 ml for bench scale and microscale vessels, respectively. The two bench scale vessels were operated at 1.0 vessel volume exchange per day (VVD). Microscale bioreactors were operated at 1.0 VVD or 1.5 VVD. The data presented are solely derived from cultures operated at 1.0 VVD to allow comparability across scales. In Experiment 2, 12 microscale bioreactors (ambr 15) were inoculated with cells in the basal media for parallel operation and from the same inoculum train, with a starting volume of 15 ml. These vessels were also operated at 1.0 VVD.

Microscale reactors operated at 1.0 VVD were allowed three partial media exchange steps per day, conducted at 8‐hr intervals. Microscale vessels operated at 1.5 VVD were allowed 4.5 partial media exchanges per day on average; four and five exchanges on consecutive days, conducted at 5‐hr intervals, with one 8‐hr interval included on the days with four exchanges, aligning with routine sampling of 1.0 VVD culture stations. All cultivations were perfused with enriched media starting from the initiation of perfusion mode, approximately 96 hr after inoculation.

Gravity cell settling was used to introduce cell retention and exchange of cell‐free culture media to facilitate the pseudo‐perfusion mode of operation in the microscale bioreactors. The bench scale vessels were operated in perfusion mode using an ATF device (Repligen, Waltham, MA) in combination with 0.22 µm hollow fibre filter for cell retention, and continuous perfusion rate of 1.0 VVD. pH and DO were monitored online in the two 7‐L STRs via probes (Mettler Toledo, Columbus, OH), and in the microscale vessels via fluorescent spot sensors (Presens, Regensburg, Germany) located on the bottom surface. pH (7.2) and DO (50%) control was maintained through CO_2_ and O_2_ sparging, respectively. Agitation was increased with increasing oxygen demand, and antifoam was added as required.

### 2.3 Microscale cell retention – liquid exchange step

Vessels (V) were arranged in positions 1–6 on each culture station (CS). Gravity cell settling was achieved by stopping gassing and agitation, allowing cells to settle in preparation for liquid exchange. Following an initial investigation (Section 3.1.1), a settling time of 30 min was allowed in all CSs in Experiment 1. Experiment 2 evaluated settling times of 33.5 and 37 min in two CSs.

Liquid was removed from the vessels in batches of <0.9 ml and the pipette tip was only minimally immersed to avoid affecting the settling of the cells. This was facilitated by tuning the software of the automated microscale system, which allowed fine control over the height at which the liquid removal pipette travels into the vessel (to the precision of 0.01 mm). The tip height was plotted against the vessel volume by weighing the remaining liquid after aspirating at different recorded heights and the depth from which the pipette sampled was determined from this plot (slope = 0.3899; y‐intercept = 0.6203, R² = 0.9987). The sequential exchange of liquid automated by the microscale controller, took 20–30 min of additional time before cells were resuspended and gassing was reinitiated.

### 2.4 Bioreactor sampling and sample analysis

All vessels were sampled daily. Viable cell counts and percentage viability (via the trypan blue exclusion method) were conducted using an automated Vi‐Cell XR cell counter (Beckman Coulter, Brea, CA). Offline metabolite analysis for glucose and lactate concentration of the samples collected from the microscale vessels and the STR vessels were carried out in YSI 2900 (Xylem Analytics, Letchworth Garden City, UK) and Bioprofile FLEX (Nova Biomedical, Waltham, MA), respectively. The amino acid content of the spent medium was determined by liquid chromatography (ACQUITY UPLC system, AccQTag Ultra C18 column; Waters), and the peaks were evaluated against commercial standards (Waters, Milford, MA). All values were scaled to maintain commercial confidentiality. The mAb concentration was determined using Protein A’dip‐and‐read’ Biosensors on the Octet System (Pall ForteBio, Fremont, CA). A serial dilution of the purified mAb product was performed and run in parallel with inter assay controls to determine the titre and control run variation. mAb aggregation was determined by SEC‐UPLC. Unpurified, clarified cell culture supernatant was filtered through a 0.22‐µm AcroPrep plate filter (Pall, New York, NY) before loading the supernatant to the automated injection by UPLC (Waters) on SEC column (Acquity UPLC Protein BEH SEC. 200 Å; Waters); peak detections were made at wavelengths 220 and 280 nm. Protein A capture purification was performed on pooled perfusate material from each experiment (microscale 1, 2A, 2B, and bench scale) and aggregate abundance was determined by SEC‐UPLC as detailed above.

### 2.5 Rate calculations

Integral viable cell concentration, specific glucose consumption, and specific net lactate change is calculated as reported elsewhere (Adams, Korke, & Hu, 2007; Villiger‐Oberbek, Yang, Zhou, & Yang, 2015).

The integral viable cell concentration (IVCC) for each culture is calculated by:

$${\mathrm{IVCC}}_{n}=\;{\mathrm{IVCC}}_{n-1}+\left(\left(\frac{{\mathrm{VCC}}_{n}+{\mathrm{VCC}}_{n-1}}{2}\right)\times \left(t_{n}-\;t_{n-1}\right)\right)$$

where VCC stands for viable cell concentration (VCC), (t) stands for time, and the subscripts (n) and (n‐1) denote consecutive sampling points.

The specific glucose consumption (q_c,n_) is calculated by:

$$q_{c,n}=\frac{\left(c_{x,n-1}\times \left(1-D_{n}\right)+\left(m_{x}\times D_{n}\right)\right)-c_{x,n}}{{∆\mathrm{IVCC}}_{n}}$$

where *D*, *m*_*x*_ and *c*_*x*_ are the daily dilution rate, measured glucose in fresh media and measured glucose concentration in culture, respectively.

The specific net change in lactate (q_l,n)_ is calculated suing measured lactate in culture (*p*_*x*_,):

$$q_{l,n}=\frac{p_{x,n}-(p_{x,n-1}\times \left(1-D_{n}\right))}{{∆\mathrm{IVCC}}_{n}}$$

No product retention occurred and thus specific productivity (q_p,n_) was calculated by:

$$q_{p,n}=\frac{{\mathrm{Titre}}_{n}}{{\mathrm{VCC}}_{n}}\times D_{n}$$

### 2.6 Data analysis

Mean and standard deviation (*SD*) are reported as (mean ± *SD*) for all replicate data. The number of replicates from a single culture station in Experiments 1, 2A, and 2B microscale setup were *n* = 6, 4, and 6, respectively. Paired or unpaired two tailed *t* tests were used, where appropriate, to determine significance across conditions and each specific application is referred in the text. Comparison from microscale to bench scale (*n* = 2) was made using one‐way analysis of variance. Vessels within a single culture station were treated as replicates for reporting statistical significance except when the vessel position effect was investigated. All raw data are provided for reference (Supporting Information Data).

## 3 RESULTS AND DISCUSSION

### 3.1 Process design and evaluation

#### 3.1.1 Settling time requirements

The settling time for the microscale cell retention was determined keeping the following design considerations in mind: (a) allowing the exchange of an almost cell‐free fraction, i.e. the perfusate, with fresh medium, (b) maximizing potential total volume exchanged per day, while minimizing the volume exchanged per cell settling to mimic the operation at larger scales, and (c) allowing an operation at industrially relevant dilution rates, whilst minimizing the effects the periodic cell settling and media exchanges.

The limiting factor in determining the number of cell settling steps in the current design was the capacity of the automated sampling station and operator requirements. Minimal operator intervention (maintenance carried only once per day) was achieved by liquid exchange using 1‐ml capacity pipette tips adjusted for use at 90% of their capacity and this was repeated three times in each exchange step. The depth at which the pipette tip was immersed to disturb the settled cells minimally was 2.3 mm below the liquid surface.

**Figure 1:**
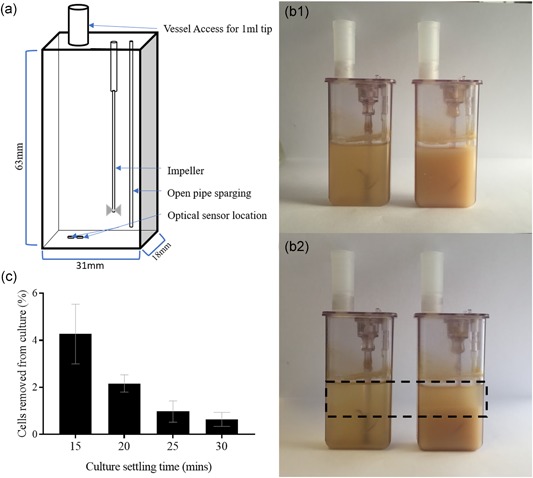
Effect of settling time and culture cell concentration on the retention of cells, and the resulting pH and dissolved oxygen (DO) effect. (a) Diagrammatic representation of microscale vessel dimensions detailing the location of optical sensors on the internal base of the vessel. Measurements for vessel geometry taken from Nienow et al (2013). (b) Photograph of two microscale vessels populated with Chinese hamster ovary cell cultures of concentrations of 2 × 10^7^ cells/ml (on the left) and 1 × 10^8^ cells/ml (on the right). The top image (B1) displays a homogenous culture at the initiation of gravity settling whilst the bottom one (B2) displays the same vessels after 30 min of settling. A sediment layer and a cleared fraction are visible in the vessels settled for 30 min indicated by the dashed box. (c) Percentage cell loss from microscale reactor vessel following cell retention by incrementally increasing the gravity settling time from 15 to 30 min, with starting concentration of 6.08 × 10^6^ cells/ml across replicates. Data show mean ± *SD*, *n* = 6 [Color figure can be viewed at wileyonlinelibrary.com]

With a simplified microscale vessel geometry by a rectangular prism (Figure 1a; 18 mm x 31 mm x 63 mm; Nienow et al., 2013), and theoretical settling velocity of 1.4 cm/h for CHO cells (Searles et al., 1994), the cells were calculated to travel 7 mm in 30 min during settling, allowing the sampling of the top 2.7 ml from the vessels. This nominally “cell‐free” supernatant was investigated further, and 99% cell retention was achieved after 30 min (Figure 1c). To preserve the concept of perfusion mimic and to avoid evaluation of a chemostat‐like environment, settling times <30 min were avoided due to low efficiency cell retention. The cells were challenged further by increasing the settling time of the CSs to 33.5 and 37 min, to comprehensively explore the effect of extended exposure of the cells to micro‐aerated and nutrient‐limited conditions on their viability (Section 3.2).

#### 3.1.2 Capacity

At 1.0 VVD and 1/3rd of the culture volume exchanged at each step, a CS operating at 50% capacity (6 vessels out of a possible 12) required additional 30 min to handle the robotic liquid exchange. Operating four CSs at 50% capacity required settling or sampling to be scheduled for 12 hr per day and at‐line measurements such as automated cell counting carried out required an additional 4 hr. The necessary daily downtime for system maintenance, when the robotic liquid handling arm was idle, was 30 min. This setup allowed us to run 24 vessels in the 48‐vessel variant of the microscale system, which would be sufficient for Design of Experiments (DoE) studies of continuous systems.

Increasing the volume exchange rate to a higher value than 1.0 VVD would limit the physical space that the cells were to settle into, thus the number of iterations of liquid exchange steps per day would need to be increased. Although the capacity of this setup was able to sustain operation at as high a volume exchange rate as 1.5 VVD, the corresponding specific growth rate (µ) of 0.062 hr^−1^ exceeded the maximum specific growth rate (µ_max_ = 0.05 hr^−1^) reported for CHO cells (López‐Meza et al., 2016). Thus, this setup was suitable for the range of all reported specific growth rates, at which CHO cells could operate.

#### 3.1.3 Resuspension of the culture and recovery to the set points

**Figure 2:**
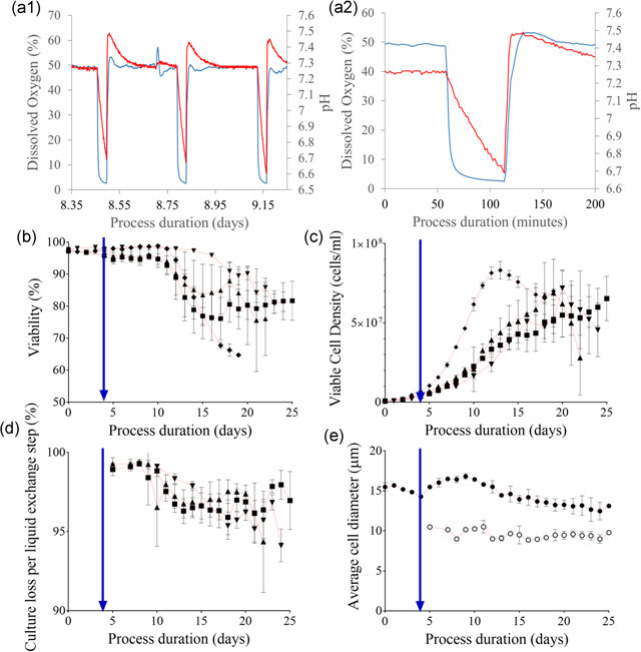
The response of the microscale system process pH and DO to cell settling, and the subsequent impact on cell growth and viability compared with bench scale operation. (a1) Sample traces from one microscale vessel in Experiment 1; inline DO (red) and pH (blue) measurements recorded during the process. Both parameters are plotted on their respective axes against the process duration (days). Variation in the acidity and oxygenation during one day of cultivation, which entails three exchange steps (1.0 VVD volume exchange rate), displayed the recovery between liquid exchange steps back to control set points. (a2) Zoomed view to one settling period from (a1) representing system response to cell settling and resuspension. (b) Viability (%) plotted against duration of cultivation (days) for the microscale vessels and the two bench scale reactors. (c) Viable cell concentration (cells/ml) plotted against process duration (days) for the microscale vessels and the two bench scale reactors. (d) Cell loss per liquid exchange step (%) determined as the ratio of the total cell content of perfusate to the total cell count for its respective culture suspended plotted against culture age. For (b)–(d): Microscale reactors: (▲) Experiment 1, *n* = 6 (30 min settling); (▼) Experiment 2, *n* = 4 (33.5 min settling); (■) Experiment 2, *n* = 6 (37 min settling); Bench scale reactors: (♦) Experiment 1, *n* = 2. Data show mean ± *SD*. (e) Average cell diameter (µm) plotted against process duration (days) for the cell samples in the microscale cultures (●) and in the perfusate (○). Plot represents the mean for all microscale samples (*n* = 16) with *SD* shown when the variation was greater than 3.5% of the mean. Perfusion initiation is indicated in each respective plot with an intersecting blue arrow. DO: dissolved oxygen [Color figure can be viewed at wileyonlinelibrary.com]

Gas sparging and agitation needed to be turned off to allow gravity cell settling, and consequently, the measurements of pH and DO were observed to rapidly decline during this period (Figure 2a1‐c). pH dropped by approximately 0.8 units over 30 min and the decline in pH started immediately after the agitation was stopped, gradually slowing down towards the end of the settling time. The DO immediately and very sharply declined from the set point (50%) down to below 5% saturation within 10 min and readings plateaued between 2 and 5% saturation. The pH and DO measurements recovered following resuspension. The drop in DO and pH was likely to be caused by the metabolic activity of the cells consuming residual oxygen and producing CO_2_ and lactate. As oxygen became limited (approximately after 10 min of cell settling; Figure 2a2), the slowing down of the cellular metabolic activity through reduced oxidative phosphorylation at low (<5%) DO saturations (Heidemann, Lütkemeyer, Büntemeyer, & Lehmann, 1998) could consequently have slowed down the rate of pH drop, since CO_2_ production would slow down.

The location of the optical sensors at the bottom of the vessel was thought to contribute to the magnitude of these observed changes (Figure 1). As cells settled down at the bottom of the vessel, the sensors only picked up the parameters associated with an oxygen depleted and by‐product accumulated local environment, which was extremely hypoxic, but not representative of the entire culture.

Hydrodynamic investigation of the mixing time within microscale vessels indicated that <5 s would be required for resuspension (Nienow et al., 2013). pH was observed to recover back to its set point immediately following resuspension, with initially a slight increase above the set point for a very short period of time. The DO rapidly responded to the reintroduction of aeration and agitation, however, the values were observed to overshoot immediately after resuspension, only to recover to the set points within the first hour. Increased sparging of oxygen into the vessel to recover the DO back to 50% was thought to remove CO_2_ from the culture, resulting in the slight elevation of the pH, an issue commonly mitigated in scale up applications (Matsunaga, Kano, Maki, & Dobashi, 2009).

### 3.2 Evaluation of the culture performance at microscale

#### 3.2.1 Viable cell concentration

Microscale vessels reached the maximum viable cell concentration of 7.20 ± 1.14 × 10^7^ cells/ml, 20 days after inoculation, whereas bench scale cultures inoculated from the same source reached the maximum viable cell concentration 13 days after inoculation and the maximum viable cell concentration was 8.32 ± 0.55 × 10^7^ cells/ml (Figure 2c). The difference in maximum viable cell concentrations of the cultures was not significant (*p* = 0.24). The exponential growth phase of the cultures growing at microscale was longer than those growing at the bench scale, and this was thought to be at least partially related to the efficiency of gravity cell settling. At microscale, the percentage of culture retained during each liquid exchange step dropped towards 95% as the cell concentration increased (Figure 2d). This minimal loss was thought to be similar to a small and frequent cell bleed that could have contributed to the depressed growth rates and lag time in reaching maximum cell concentration. In contrast, cells were considered to be retained at 100% efficiency (with no bleeding) at the bench scale as the hollow fibre filter pore size was sufficiently small to prevent the passage of cells.

Increasing the settling time from 30 to 33.5 min did not yield a significant change (*p* = 0.91) in the maximum viable cell concentration, although the average maximum value attained was slightly lower, 6.88 ± 2.13 × 10^7^ cells/ml. Further increase in retention time to 37 min did not yield a significant change (*p* = 0.38 and 0.45, respectively) in maximum viable cell concentration either, although the average concentration was lower than that for 30 and 33.5 min, 6.53 ± 1.40 × 10^7^ cells/ml. There was a variation in the time taken to reach maximum viable cell concentration in Experiment 2A, with the average 19.5 ± 1.73 days being slightly earlier than the 20 days after inoculation observed for all replicates in Experiment 1. However, this difference was not significant either (*p* = 0.49). Vessels across Experiment 2B did not reach this maximal cell concentration until much later (Day 25), and this difference was highly significant (*p* < 0.001).

A nonsignificant decrease (*p* = 0.15) was observed in cell viability (Figure 2b) on the day of the maximum viable cell concentration as the settling time was increased from 30 min (90.2 ± 2.0%) to 33.5 min (86.9 ± 4.5%). The decrease in cell viability was not significant (*p* = 0.18) when the settling time was further increased from 33.5 min (86.9 ± 4.5%) to 37 min (81.7 ± 6.1%). However, the total decrease in viability observed with increasing the settling time from 30 min to 37 min was statistically significant (*p* < 0.01), suggesting an overall adverse effect of increasing the culture settling time on viability.

#### 3.2.2 Culture retention and perfusate analysis

Cell content of the perfusate was determined to evaluate the retention performance of the microscale experiments (Figure 2d). Average cell retention on the day of maximum viable cell concentration was 96.2 ± 0.5, 97.5 ± 0.5, and 97.0 ± 1.8%, for Experiments 1, 2A, and 2B, respectively. These values were higher than the values reported earlier (90% retention; MilliporeSigma, 2017) indicating improved performance in the present setup.

As cultures progressed into the late exponential growth phase (18–21 days after inoculation), Experiment 2 cultures with extended settling times displayed a statistically significant increase in average cell retention (*p* < 0.05). No significant difference was observed between the two CSs with 33.5 and 37 min retention times in Experiment 2.

The average diameter of the cells across all microscale culture samples and microscale perfusate samples was 13.27 ± 0.95 and 9.50 ± 0.50 µm, respectively (*n* = 16; across 4 days [18–21]). The difference between these measurements was highly significant (*p* < 0.001; Figure 2e).

Viability of the perfusate samples at maximum viable cell concentration (93.1 ± 4.8%) was consistently higher than that of the homogenous microscale cultures (86.2 ± 5.5%; *n* = 16, *p* < 0.001). Although cell viability was reported to affect settling velocity in early reports (Searles et al., 1994), with nonviable cells moving 30–50% slower than viable cells (Wang & Belovich, 2010), this was not observed in the cultures investigated here. However, given the difference in size, the cells removed were more likely those, which displayed lower specific productivity due to their stage in cell cycle, as reported earlier (Lloyd, Holmes, Jackson, Emery, & Al‐Rubeai, 2000).

#### 3.2.3 Variability within and across culture stations, and across inoculum trains

The microscale vessels were serviced sequentially by the robotic arm during perfusate‐fresh medium replacement, and this introduced additional lag time for each vessel placed at positions 2–6 on each CS (Figure S1C).

Viable cell concentrations were compared across positions to identify any position‐specific variation in settling time across and within CSs (Figure S1). Experiments 1 and 2A were evaluated as biological replicates, and Experiments 2A and 2B as technical replicates. There was no clear trend displaying significant differences in both biological replicates, although some significant positional effects were observed between the neighboring vessels in each CS (Figure S1A,B). Increasing the settling time to 37 min caused significant variability across subsequently serviced vessels (Figure S1B). In line with these observations, the suggested total settling and servicing time of each vessel needed to be shorter than 41 min for this cell line. Although this observation will be cell line specific, the range of operation investigated in this study provides a sufficiently wide investigation space to select to operations range from.

#### 3.2.4 Controlled process parameters and biochemical analysis

**Figure 3:**
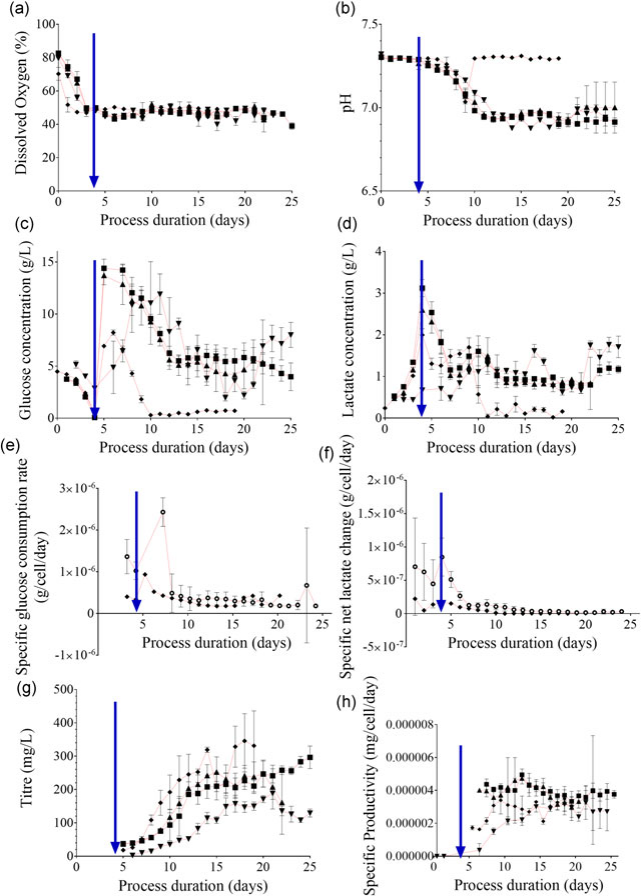
Culture response in online and offline measured process parameters to the mode and the duration of cell retention. Online microscale vessel readings have been averaged over their respective 24‐hr periods to provide a representative value for the day. All parameters are plotted against process duration (days). (a) DO (%), online (b) pH, online (c) Glucose (g/L), offline (d) Lactate (g/L), offline (e) Specific glucose consumption rate (g/cell/day), (f) Specific net lactate change (g/cell/day), (g) Titre (mg/L), offline (h) Specific productivity (mg/cell/day). For (a)–(h): Microscale reactors: (▲) Experiment 1, *n* = 6 (30 min settling); (▼) Experiment 2, *n* = 4 (33.5 min settling); (■) Experiment 2, *n* = 6 (37 min settling); (**○**) combined microscale from Experiment 1 and 2, *n* = 16; (♦) Bench scale reactors: Experiment 1, *n* = 2. Data show mean ± *SD*. Perfusion initiation is indicated in each respective plot with an intersecting blue arrow. DO: dissolved oxygen [Color figure can be viewed at wileyonlinelibrary.com]

The microscale system was able to supply sufficiently large volumes of oxygen to support the culture growth even at the high viable cell concentrations observed, as was the case for the bench scale system (Figure 3a). The pH of both microscale and bench scale cultures got slightly more acidic but were maintained within the control set points for 7–10 days after inoculation (Figure 3b). The culture pH further became more acidic from this day onward. The bench scale systems recovered back to the initial set point, whereas the pH of the microscale vessels consistently remained slightly more acidic at 6.9.

Glucose was gradually consumed by the cultures before perfusion being initiated at 4 days after inoculation (Figure 3c). Once perfusion was initiated, the influx of enriched media increased culture glucose concentrations, and glucose was increasingly utilized as viable cell concentrations increased. Glucose was available for all cultures in microscale vessels on the day of maximum viable cell concentration; 2.22 ± 0.37, 3.07 ± 1.02, and 3.98 ± 1.33 g/L, respectively, for Experiments 1, 2A, and 2B, indicating that the cultures were not glucose‐limited. The lactate accumulated during the batch phase of the microscale cultures was 0.67 ± 0.05, 2.59 ± 0.48, and 3.12 ± 0.20 g/L for Experiments 1, 2A, and 2B, respectively (Figure 3d). The lactate concentration remained similar throughout the perfusion phase.

In contrast, glucose was fully utilized in the bench scale cultures, with the remaining glucose determined as 0.51 ± 0.04 g/L on the day of maximum viable cell concentration. The glucose concentration dropped from a peak at the 6th day after inoculation (8.22 ± 0.25 g/L), and was fully utilized starting from the 10th day after inoculation (0.29 ± 0.01 g/L). A metabolic shift was observed as the cultures also metabolized the available lactate, dropping from 1.70 ± 0.23 g/L on the 9th day after inoculation, to 0.04 ± 0.06 g/L by the 11th day after inoculation. Utilization of lactate increased the pH, recovering the pH back to the initial set point in these cultures. This metabolic switch did not occur in any of the microscale vessels and consequently, the pH continued to drop as discussed above.

A significant difference (*p* < 0.05) was observed in the glycine, alanine, glutamine, and glutamate pools between the bench scale and microscale cultures starting from the 12th day after inoculation. The depletion of glucose to 0.29 ± 0.14 g/L on the 10th day following inoculation was coupled with the utilization of lactate for pyruvate production, which would then proceed into the citric acid (TCA) cycle for the generation of energy. Pyruvate would selectively be converted into α‐ketoglutarate, which enters the TCA cycle, and excess pyruvate would be converted into glycine (Figure S2C). The concurrent increase in the by‐product alanine concentration provided further support for the activation of this pathway (Figure S2B). Glutamine concentration was shown to decrease (Figure S2A) indicating that the glutamate pool was utilized in α‐ketoglutarate production, rather than glutamine.

On the other hand, asparagine, cysteine, and serine were shown to concurrently deplete over time for both bench scale and microscale cultures (Figure S2D–F). The concentration of the other amino acids remained constant, with slight statistically insignificant variations (*p* > 0.05; Figures S3 and S4).

Despite these differences, the specific glucose consumption and net specific lactate production (difference between production and back‐metabolization) of the microscale and bench scale systems were similar (Figure 3e–f). Earlier reports indicated that even a small variation in pH as 0.1 could significantly impact culture growth and metabolism, in particular glucose consumption and lactate production (Li, Vijayasankaran, Shen, Kiss, & Amanullah, 2010). The cell line used in this study was very robust, withstanding a pH variation of 0.3 without demonstrating any observable differences in two major indicators of metabolic activity after the 10th day following inoculation. For sensitive cell lines, the variation in pH can be confined to as small a range as needed by implementing two‐point control in the microscale system.

#### 3.2.5 Productivity and product quality

On the day of the maximum viable cell concentration, the product concentration was 189 ± 19, 267 ± 18, and 267 ± 13 mg/L for microscale Experiments 1, 2A, and 2B, respectively (Figure 3g). The differences observed between Experiments 1 and 2A, as well as between Experiments 1 and 2B, were significant (*p* < 0.001 for both). The product concentration in the bench scale vessels was 253 ± 9 mg/L at the point of maximum viable cell concentration and the difference between the microscale and the bench scale product concentration was not significant (*p* = 0.92).

The variation in cell‐specific productivity across cultures was observed to decrease as the cultures approached similar phases of operation (Figure 3h). The cultures were more uniform with less variation towards the end of the process as implicated by their average specific productivity (3.05 ± 1.1 × 10^6^ mg/cell/day on the 8th day after inoculation vs. 3.06 ± 0.17 × 10^6^ mg/cell/day on the 19th day after inoculation, *p* = 0.86). This indicated that microscale perfusion was a robust mode of operation for assessing productivity.

Protein A‐purified perfusate samples collected from the vessels at the time of maximum viable cell concentration were assessed for the purity of the monomer mAb for all operations. The product purity achieved by cultivation at microscale was 93.3, 95.1, and 94.0% for Experiments 1, 2A and 2B, respectively, and that achieved at bench scale was 94.0%, indicating comparability across different scales of operation.

Furthermore, the average monomer content of the perfusate was also determined before Protein A purification. This “raw” quality measurement indicated by the monomer content was 61.0 ± 0.7, 54.6 ± 8.7, and 53.3 ± 6.5%, for the microscale Experiments 1, 2A, and 2B, respectively. The differences observed between Experiments 1 and 2A and between Experiments 2A and 2B were not statistically significant (*p* = 0.1 and 0.8, respectively). However, the product quality was significantly lower in Experiment 2B than in Experiment 1 (*p* < 0.05). The long settling times could have caused reduced product quality. Nevertheless, these measurements were all higher than the monomer content of the bench scale cultures on the 14th day after inoculation (44.8 ± 0.7%), and this difference between the bench scale and microscale cultures was highly significant (*p* < 0.001, compared to Experiment 1). Despite the variation observed in Experiment 2, a settling time longer than 40 min was still well‐tolerated by this cell line, high quality product could still be achieved. The microscale perfusion mimicking setup did not negatively impact product quality as ATF bench scale perfusion environments were reported to do so (W. Kelly et al., 2014; Walther, McLarty, & Johnson, 2019).

These analyses were extended to evaluate the performance of two additional cell lines grown in microscale perfusion as detailed in this study and also to compare this with their performance in bench scale reactors operating at their cell‐specific perfusion rate. No significant difference was observed in specific productivity (*p* = 0.88) or culture performance between the microscale and bench scale modes of operation, providing additional support for the broad applicability of the approach.

## 4 CONCLUSIONS

In this study, the design and performance of a microscale bioreactor system operating under pseudo‐perfusion mode was explored, with several considerations outlined (Figure S5). Gravity settling was used to facilitate cell retention, and a settling period longer than 30 min was required for effective cell retention, where 95% of the cells were settled before liquid exchange, and that the upper limit for cell settling period should be closer to, but shorter than, 41 min to maintain viability above 86% for prolonged cultivation periods.

The microscale system supported statistically comparable maximum cell concentration displayed in bench scale operation specific for this experimental cell line, with cultures reaching very high cell concentrations exceeding 7.0 × 10^7^ cells/ml without being challenged by oxygen limitation or increased acidity of the culture environment. The slight differences in culture pH between the microscale and bench scale operations did not yield differences in the CHO cell physiology, with the specific glucose consumption and net specific lactate production rates remaining similar.

The present setup could utilize either the 48‐ or the 24‐vessel variants of the microscale system at 50% capacity with half of each culture station being utilized at a time. At a liquid exchange rate of one vessel volume per day, this setup allowed suitable maintenance downtime and a single operator intervention per day. Our evaluation has shown the potential and applicability of this setup in conducting multifactorial DoE to effectively evaluate perfusion process set points, media optimization or as a cell line screening tool.

## Supporting information

Supporting information

Supporting information

Supporting information

Supporting information

Supporting information

Supporting information

Supporting information
