# Supplementary figures and images for: Enhancing the functionality of a microscale bioreactor system as an industrial process development tool for mammalian perfusion culture
Source: Biotechnol Bioeng. 2019 Feb 20;116(6):1315–25. doi: 10.1002/bit.26946 (PMC6593443; doi:10.1002/bit.26946)

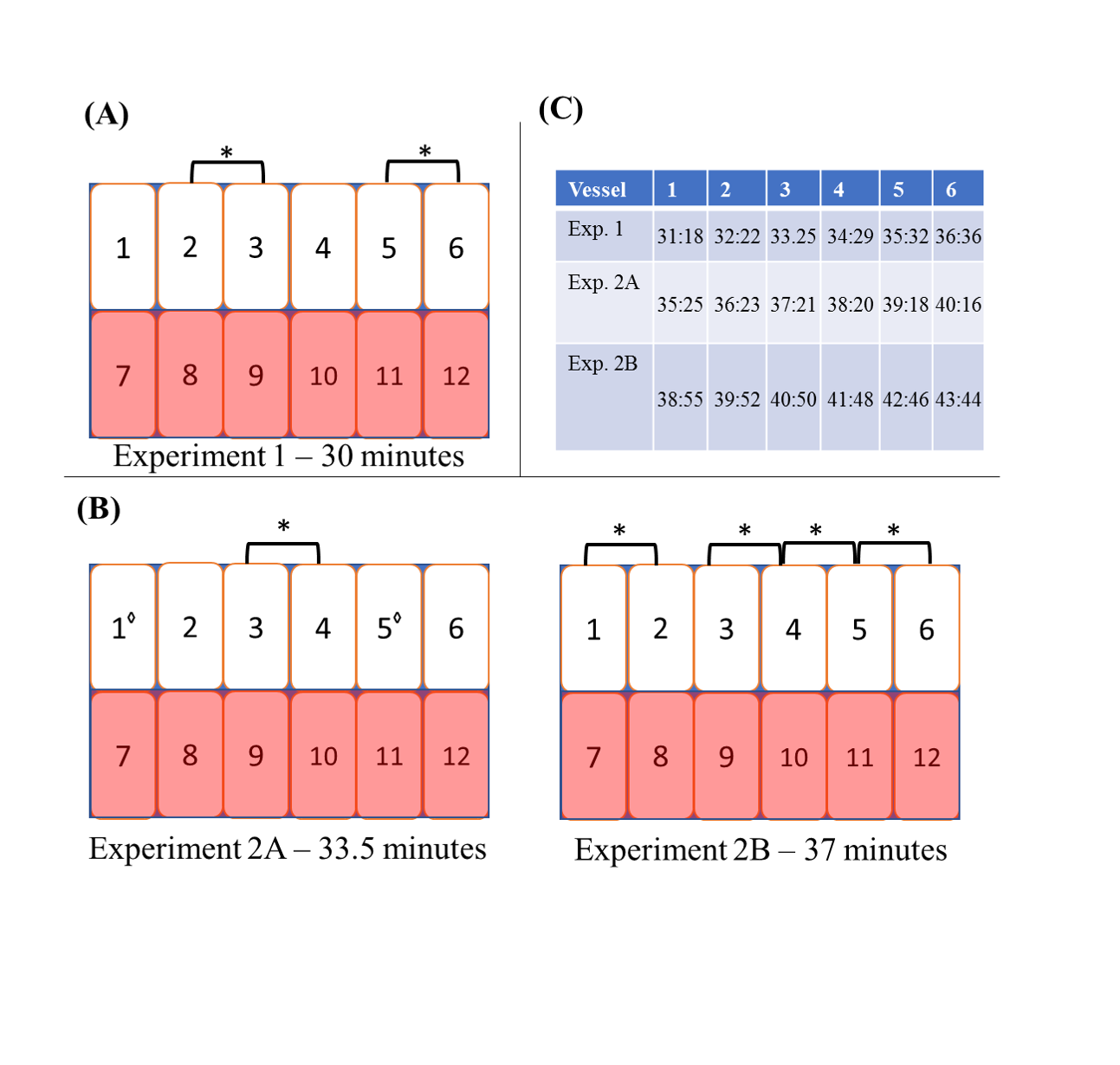

Supplement: Supplementary file 1 — Supporting information [file BIT-116-1315-s001.tif]

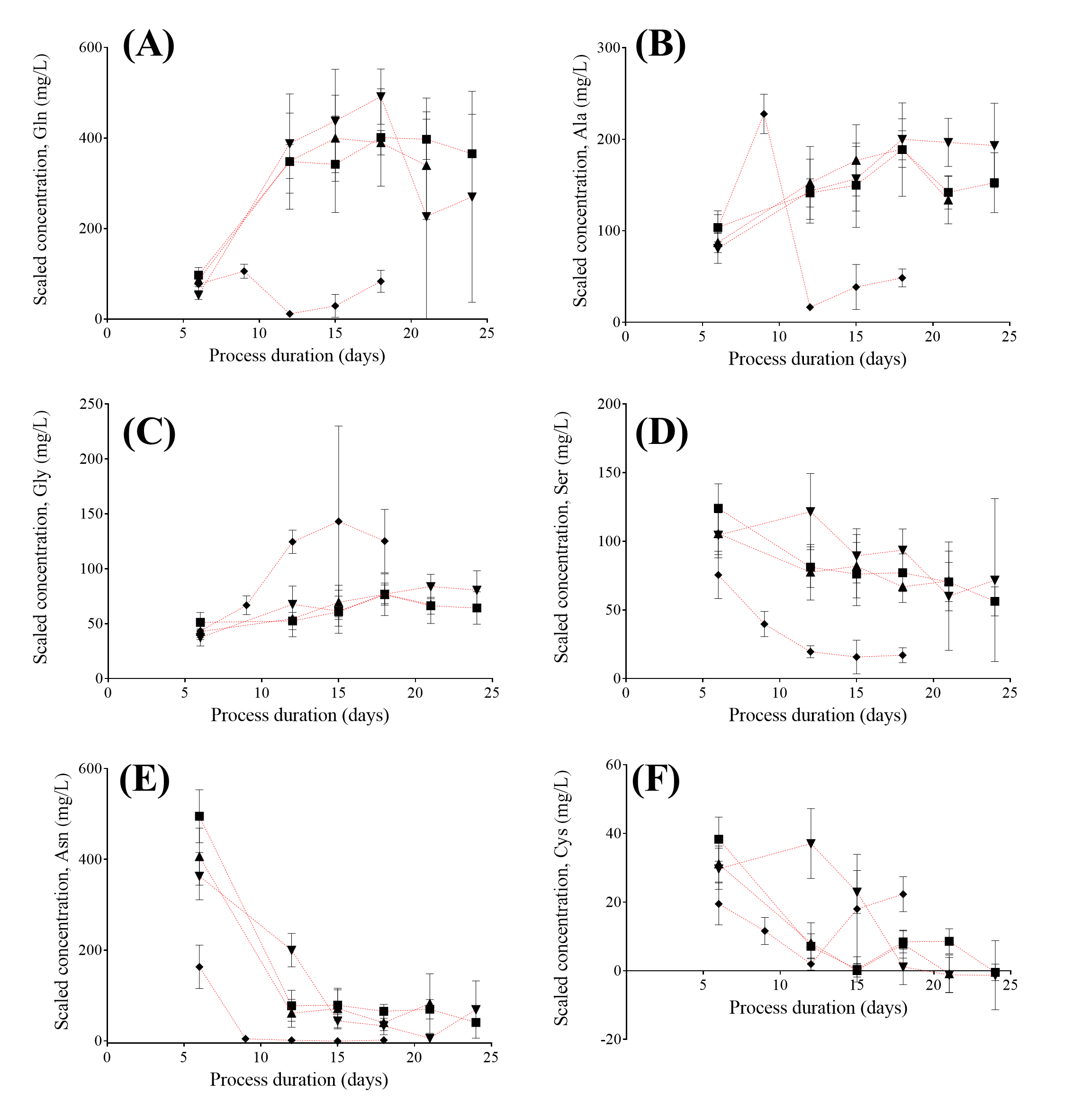

Supplement: Supplementary file 2 — Supporting information [file BIT-116-1315-s002.tif]

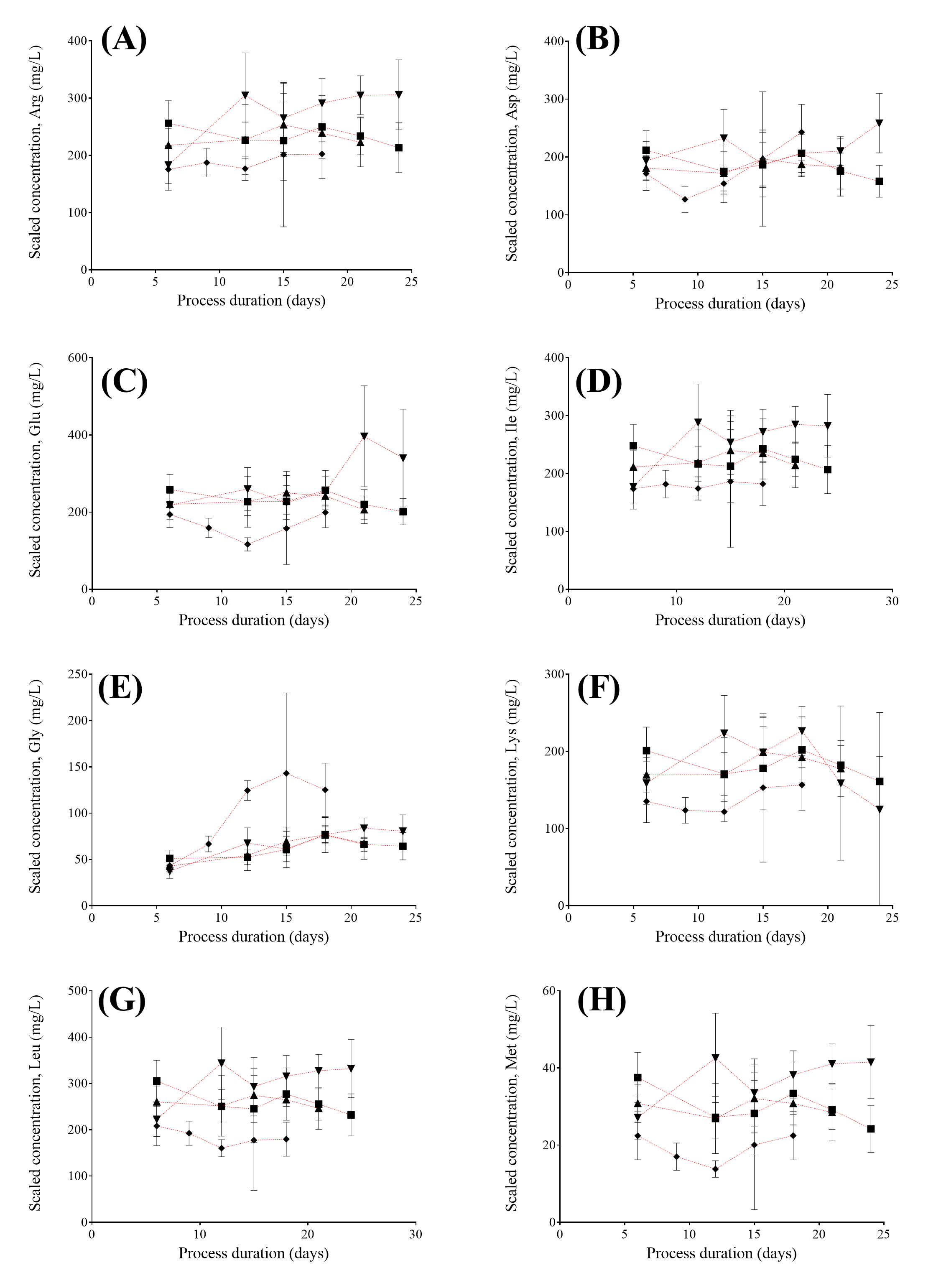

Supplement: Supplementary file 3 — Supporting information [file BIT-116-1315-s003.tif]

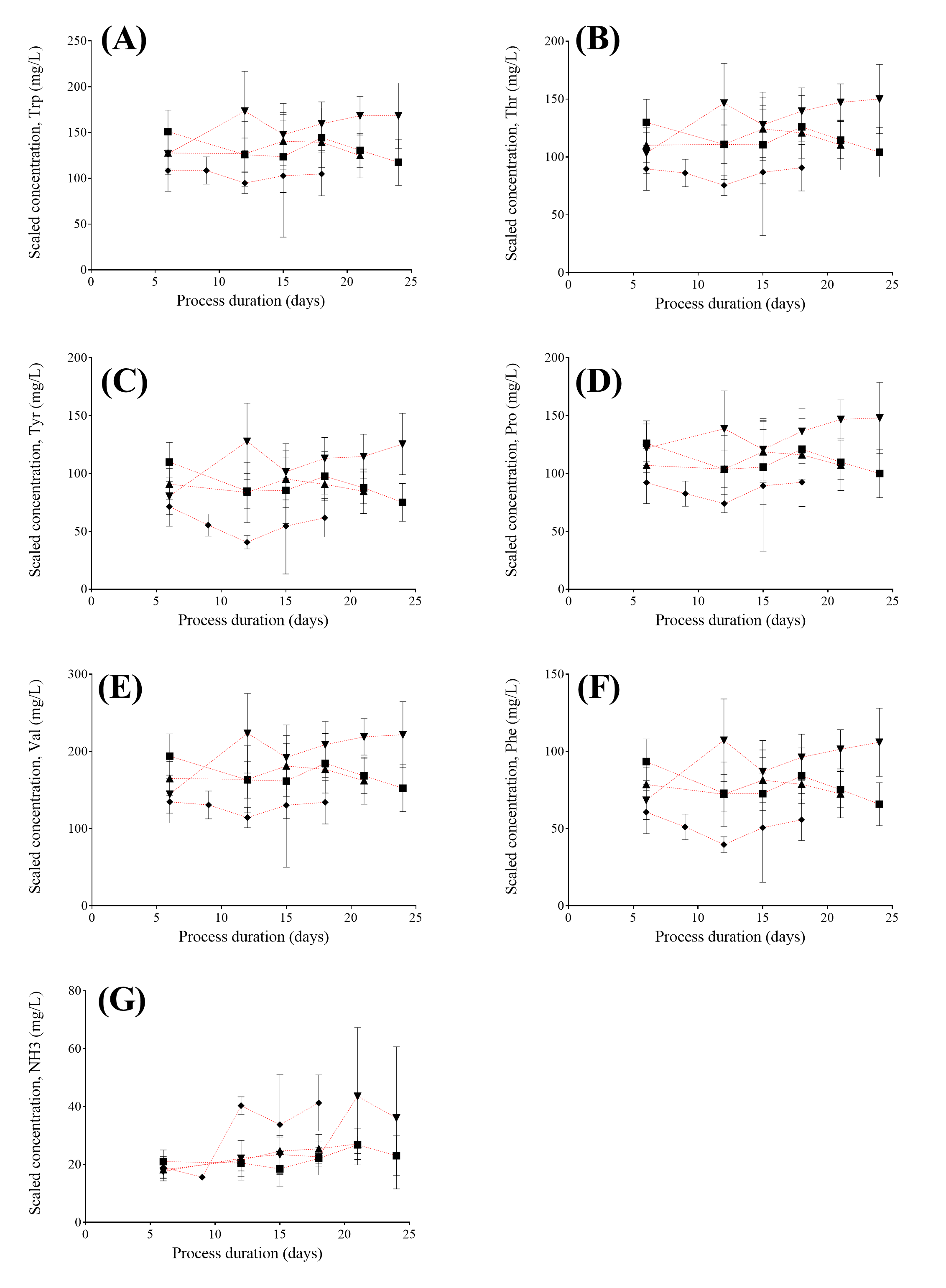

Supplement: Supplementary file 4 — Supporting information [file BIT-116-1315-s004.tif]

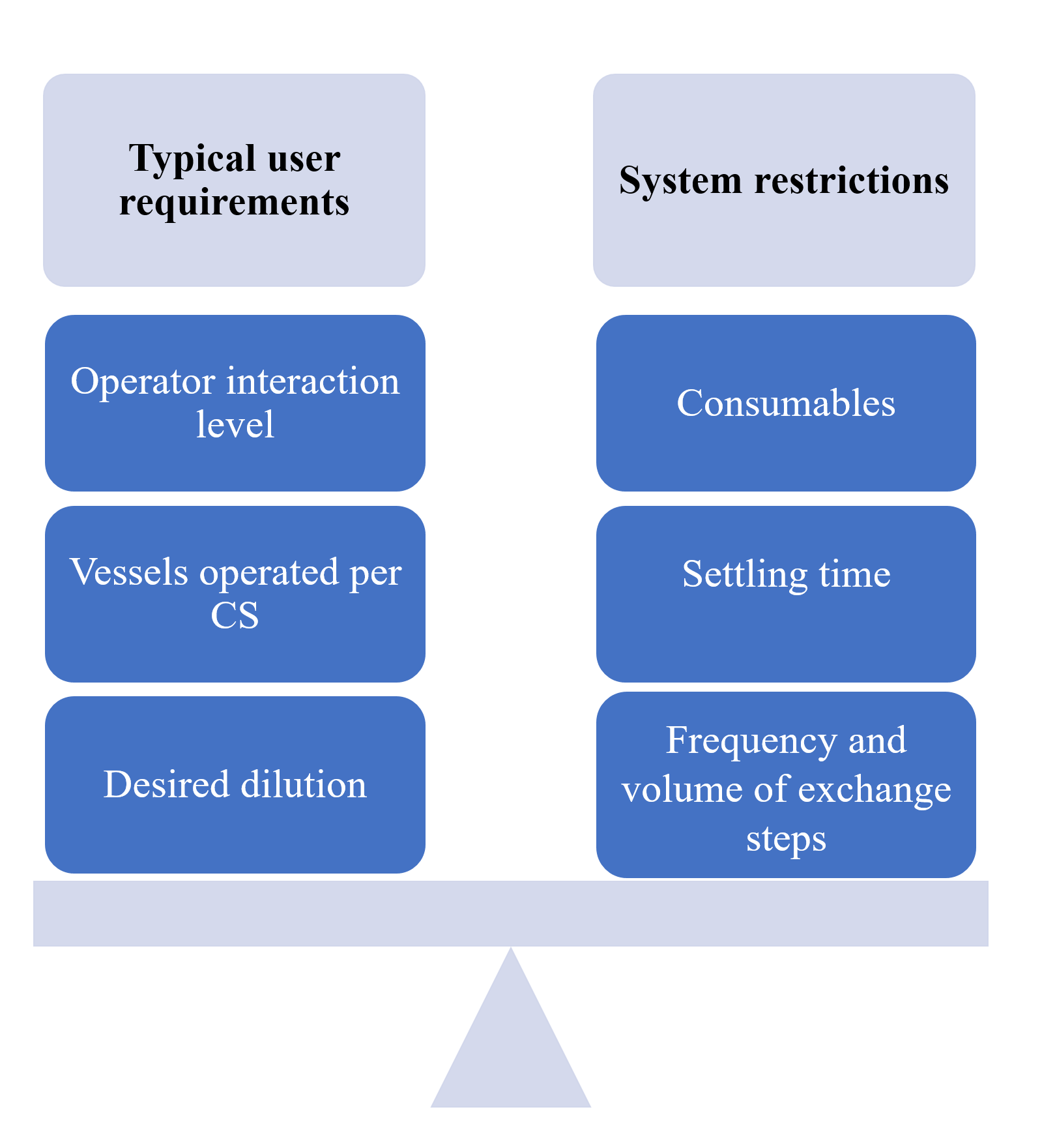

Supplement: Supplementary file 5 — Supporting information [file BIT-116-1315-s005.tif]
