# Supplementary material for: Enhancing the functionality of a microscale bioreactor system as an industrial process development tool for mammalian perfusion culture
Source: Biotechnol Bioeng. 2019 Feb 20;116(6):1315–25. doi: 10.1002/bit.26946 (PMC6593443; doi:10.1002/bit.26946)
Supplement: Supplementary file 7 — Supporting information [file BIT-116-1315-s007.docx]

**List of Supplementary Figures**

**Supplementary Figure 1. Position of the microscale vessels within their respective culture stations, and the actual settling times for each vessel observed prior to automated robotic liquid exchange.** (A) – (B) Microscale vessel location on each culture station in Experiments 1 and 2. Differences in the viable cell concentration of neighbouring vessels determined by paired t-test at a significance threshold of (P<0.05), denoted by *, where significant. (C) Actual settling times (minutes:seconds) for each vessels in their respective positions 1-6 on the CSs to the initiation of automated liquid exchange, caused by the robotic arm servicing one vessel at a time. **^◊^** Vessels 1 and 5 in experiment 2A were terminated on Day 10 due to hardware failure.

**Supplementary Figure 2. Perfusate amino acid profiles for microscale and benchscale experiments.** Figure shows the Glutamine, Alanine, Glycine, Serine, Asparagine and Cystine concentration profiles, denoted by (A-F), respectively, starting from the 6^th^ day across process duration starting at day 6 post inoculation measured every three days. Concentrations were scaled randomly in the interest of protecting the IP of the proprietary media and are reported as “Scaled concentrations (mg/L)”. Microscale reactors: (▲) Experiment 1, n=6 (30 minutes settling); (▼) Experiment 2, n=4 (33.5 minutes settling); (■) Experiment 2, n=6 (37 minutes settling); (♦) Bench scale reactors: Experiment 1, n=2. Data shows mean ± SD

**Supplementary Figure 3. Perfusate amino acid profiles for microscale and benchscale experiments**. Figure shows the Arginine, Aspartic acid, Glutamate, Isoleucine, Glycine, Lysine, Leucine and Methionine profiles, denoted by (A-H), respectively, starting from the 6^th^ day post inoculation measured every three days. Concentrations were scaled randomly in the interest of protecting the IP of the proprietary media and are reported as “Scaled concentrations (mg/L)”. Microscale reactors: (▲) Experiment 1, n=6 (30 minutes settling); (▼) Experiment 2, n=4 (33.5 minutes settling); (■) Experiment 2, n=6 (37 minutes settling); (♦) Bench scale reactors: Experiment 1, n=2. Data shows mean ± SD

**Supplementary Figure 4. Perfusate amino acid profiles for microscale and benchscale experiments** Figure shows the Tryptophan, Threonine, Tyrosine, Proline, Valine, Phenylalanine and NH_3_ profiles, denoted by (A-G), respectively, starting from the 6^th^ day post inoculation measured every three days. Concentrations were scaled randomly in the interest of protecting the IP of the proprietary media and are reported as “Scaled concentrations (mg/L)”. Microscale reactors: (▲) Experiment 1, n=6 (30 minutes settling); (▼) Experiment 2, n=4 (33.5 minutes settling); (■) Experiment 2, n=6 (37 minutes settling); (♦) Bench scale reactors: Experiment 1, n=2. Data shows mean ± SD.

**Supplementary Figure 5. Operator considerations to be taken into account when designing an effective, investigative process in adapted microscale systems.** Limitations of the microscale system place certain design considerations into the hands of the operator when adapting the system for perfusion. These culminate in evaluating what level of operator interaction is possible; increasing/decreasing the number of times consumables are refreshed each day may enable or prevent the desired volume exchange rates. Also, the number of vessels per culture station needed will impact resources and could inflict variation in total culture station settling times. Given the established requirements and restrictions, the operator has to determine the optimal setup based on the volume replacement per each step and the frequency of these steps, which is realistically attainable within their framework.

**Supplementary data**. Provided (organised in the following 7 tabs of the document ‘Supplementarydata.xlsx’), is measured data presented in this article; ‘Development fig 1 counts’, ‘Microscale DO and pH’, ‘Culture cell counts’, ‘Perfusate cell counts’, ‘% Cell retention and diameters’, ‘pH daily monitoring’, ‘Glucose and Lactate’, ‘Scaled amino acids’ and ‘Titre and Monomer’.
